# AI-Assisted Adaptation of the Swedish Version of the Digital Competencies for Applied Psychological Practitioners Scale: Preliminary Psychometric Evaluation

**DOI:** 10.2196/92332

**Published:** 2026-09-08

**Authors:** Kristofer Vernmark, Simon Lai, Alesia Moulton-Perkins, Helen Pote

**Affiliations:** 1 Department of Behavioural Sciences and Learning, Linköping University, Campus Valla, Olaus Magnus väg 37, Linköping, Östergötland, 581 83, Sweden, 46 733211074; 2 Department of Psychology, Royal Holloway University of London, London, United Kingdom; 3 Oxford Institute of Clinical Psychology Training and Research, Oxford Health NHS Foundation Trust, Oxford, United Kingdom

**Keywords:** digital mental health, clinical psychology, psychologists, psychology students, digital competencies, instrument translation, cross-cultural adaptation, psychometrics, AI

## Abstract

**Background:**

As digital technologies become increasingly integrated into psychological practice, the demand for competencies in digital clinical psychology is growing. Although competency frameworks for digital clinical practice exist, validated instruments to assess these competencies remain scarce. In Sweden, psychology master’s students are now being offered digital psychology courses, increasing the need for instruments to measure intended improvements in knowledge and abilities. Using AI to assist with translation procedures can facilitate the adaptation of existing instruments to new national and cultural contexts.

**Objective:**

This study aims to test an AI-assisted procedure for the translation and contextual adaptation of the Digital Competencies for Applied Psychological Practitioners (DCAPP) scale into Swedish and to examine the psychometric properties of the translated version in a sample of psychology master’s students in Sweden, including a pilot test of the instrument’s responsiveness to changes in knowledge and abilities among students attending a digital psychology course.

**Methods:**

An AI-assisted adaptation procedure, using ChatGPT and DeepL, was used to translate the DCAPP from English to Swedish. The Swedish version was distributed to psychology master’s students during their eighth semester, including those attending an elective digital psychology course. Twenty-four of 55 master’s students completed the baseline questionnaire. Of the 14 students enrolled in the digital psychology course, 13 completed the baseline questionnaire and 9 completed the follow-up assessment. Item descriptives, internal consistency, and responsiveness to change were calculated for the scale.

**Results:**

The AI-assisted translation procedure resulted in a translated version of the scale with both high quality and semantic similarity ratings. The Swedish DCAPP demonstrated excellent internal consistency for the total score (α=.96) and also for Knowledge (α=.93) and Abilities (α=.96) subscales. It demonstrated acceptable item distributions, with item-total correlations above 0.30 (range: 0.53‐0.87), and mean interitem correlations for the subscales were acceptable but indicated potential item redundancy (Knowledge *r*=0.48; Abilities *r*=0.61). Although skewness and kurtosis values were mostly acceptable, pronounced floor effects were observed in both subscales. A statistically significant increase in students’ competency ratings was observed at post test (*P*<.001), suggesting preliminary sensitivity to change.

**Conclusions:**

Using an AI-assisted adaptation procedure to support translation is feasible. The Swedish DCAPP showed promising psychometric properties and preliminary evidence of responsiveness to change. Floor effects may have been due to students’ limited digital competencies. Although initial results are promising, further research with larger samples is needed before the psychometric validity of the Swedish DCAPP can be confirmed.

## Introduction

### Background

Using digital technology to provide psychological treatment is not a new phenomenon [1,2]. However, the use of digital interventions substantially increased during the COVID-19 pandemic [3,4], and recent developments in AI are increasingly being applied in the treatment of mental health problems [5-7]. Digital mental health interventions such as internet-based treatments and videoconferencing have shown comparable effects to face-to-face alternatives [8,9]. Evidence suggests that human support is vital for increasing compliance and enhancing outcomes, as unsupported digital interventions are typically less effective [10-12], and a strong therapeutic alliance can be formed in digital and blended interventions [13,14]. Therefore, the role of health care professionals is crucial for achieving favorable outcomes in digital clinical interventions.

Research on the transition from in-person to remote digital clinical services has shown that therapists experience both challenges and advantages related to topics such as clinical usefulness, flexibility, use of technology, and perceived treatment quality and efficiency [15,16]. While digital delivery may be perceived as a barrier to establishing a therapeutic relationship [8], certain aspects, such as text-based interactions with patients, can make therapy less emotionally demanding for health care professionals [17]. A survey on videoconferencing during COVID-19 in Sweden also showed that cognitive behavioral therapy (CBT) clinicians perceived certain treatment components, such as role-play and interpersonal skills training, as less suitable for digital formats, suggesting that some interventions may require specific adaptations and training when delivered digitally [18]. Unfortunately, research is currently limited regarding desirable therapist behaviors for delivering services digitally, and there are few validated instruments to measure digital mental health competencies [19,20]. This is particularly the case for clinical psychologists and psychological practitioners whose roles require a broad range of skills beyond direct psychological interventions and specific formats of delivery, such as data collection, service development, supervision, and leadership, all areas affected by digitalization but not specifically addressed in psychotherapy or counseling training programs [21].

The ongoing digitalization of mental health interventions, accelerated by the COVID-19 pandemic, is changing the psychology profession and the everyday work of clinical psychologists [22,23]. Although surveys have shown that psychologists are positive toward the use of digital and hybrid formats [24], and there are examples of onboarding and training programs for psychologists [25], the actual transition to digital work has often taken place without specific training, and digital mental health competencies have been neglected in the curriculum of psychology training programs [16,26]. One example of an existing framework for practitioners is the Digital Competencies for Psychological Professions framework [27,28] developed in the United Kingdom and adopted by the British Psychological Society [21]. It is used to develop digital competencies within National Health Service–funded clinical training programs and for CBT therapists working in Talking Therapies services [29,30]. The framework consists of 8 domains, each containing core and advanced abilities and knowledge areas. It was developed by an expert reference group that included UK academics, clinical psychologists, psychological practitioners, trainers, experts by experience, digital mental health companies, and commissioners [27,28].

To measure the components (knowledge and abilities) of the framework, the Digital Competencies for Applied Psychological Practitioners Scale (DCAPP) [31] was developed by researchers at Royal Holloway, University of London. However, the instrument was developed for a UK setting, in English, making it difficult to use in other national contexts without translation and adaptation. There are well-established guidelines for the translation and adaptation of instruments and questionnaires that lay out specific steps in the translation process and considerations for retaining semantic similarity and contextual fit [32]. As translation procedures using bilingual experts and human translators are time- and resource-intensive, and the development of large language models (LLMs) and AI has accelerated, new AI-assisted translation and adaptation procedures for cross-cultural translation of questionnaires have been developed [33-35]. These approaches can simplify and speed up translation procedures without loss of quality and can be observed as a groundbreaking development in the metric-based quality assessment of translation [36].

### Objectives

The objective of this study was to test an AI-assisted translation and adaptation procedure of the DCAPP instrument for a Swedish context, gather preliminary data on the psychometric properties of the instrument, and examine its responsiveness to change when used in a sample of psychology master’s students attending a digital psychology course.

## Methods

### Participants and Procedures

The Checklist for Reporting Results of Internet E-Surveys (CHERRIES) was used to guide the reporting of the survey [37]. A completed version of the checklist can be found in Checklist 1. Data were collected between April and May 2025 using convenience sampling. Participants were fourth-year students in the 5-year professional psychology master’s program at Linköping University. During the eighth semester of the program, students could choose among 4 elective courses, each worth 7.5 ECTS (European Credit Transfer and Accumulation System) credits. One of these courses was *Digital Psychology: Research and Clinical Applications*, which was selected by 14 students. Information about the study was distributed via email to all 55 psychology master’s students in their eighth semester and via the digital psychology course website prior to the start of the course. All students were asked to complete the baseline questionnaire. Follow-up questionnaires were collected after the digital psychology course had ended and students in the course had completed their summative assessments. Two reminder emails were sent at each measurement point. Of the 55 master’s students, 24 completed the baseline questionnaire. Of the 14 students who had enrolled in the digital psychology course, 13 completed the baseline questionnaire, and 9 of those 13 students completed the follow-up assessment. All baseline responses were included in the psychometric evaluation of the Swedish DCAPP. Participants who completed both the baseline and follow-up assessments were eligible for inclusion in the responsiveness analyses, as described in the *Statistical Analysis* section.

### DCAPP

The DCAPP scale is a self-report instrument designed to assess the digital readiness of psychological practitioners. The DCAPP was developed in the United Kingdom based on the Digital Competence Framework for Psychological Practitioners, which was developed by the Division of Clinical Psychology of the British Psychological Society [27,28]. The DCAPP includes 2 subscales: Knowledge Competencies (15 items) and Abilities Competencies (16 items). Both subscales aim to measure a set of domains relevant to safe, ethical, and effective digital psychological practice. Respondents rate each item on a 5-point Likert scale, ranging from 1 (Novice; no digital knowledge or skills) to 5 (Expert; does not require further training). No items require reverse scoring. Scores are calculated separately for the Knowledge and Abilities subscales by summing the item scores within each subscale. The total DCAPP score is calculated as the sum of all items, with higher scores indicating greater digital competence. The measure has been piloted in the United Kingdom with psychological practitioners, and unpublished data suggest good reliability and face validity (Pote H, Moulton-Perkins A, Rides G, unpublished data, 2025).

### Questionnaire Design

The baseline questionnaire included the Swedish DCAPP 31-item scale and 11 additional questions on age, gender, previous digital training within the psychology master’s program or elsewhere, previous experience delivering digital formats, and the perceived necessity of digital competencies for clinical psychologists. The follow-up questionnaire included the DCAPP scale, as well as a question about whether respondents had received any additional training since starting the digital psychology course. Participants were also invited to provide feedback on each of the 31 DCAPP items, the comprehensibility of the Likert scale, and whether the knowledge and abilities items were perceived as distinct constructs. Baseline and follow-up questionnaires (excluding DCAPP questions) can be found in Multimedia Appendices 1 and 2. The survey was distributed through the Iterapi platform, a secure and widely used solution for providing online surveys, questionnaires, and internet-based interventions [38]. A detailed description of the Iterapi platform can be found in Multimedia Appendix 3.

### Translation Process

We employed an AI-assisted procedure to translate the DCAPP from English to Swedish. The procedure combined neural machine translation (DeepL) with GPT-4.0–generated translation quality evaluations, which, in a previous study, was found to produce translations comparable to those obtained using conventional translation procedures [33]. This process included using GPT Estimation Metric Based Assessment–Direct Assessment (GEMBA-DA) and custom Semantic Similarity Assessment (SSA) scores to assess the translation quality and degree of semantic equivalence between original and translated items. GEMBA-DA is an LLM-based evaluation approach in which the model directly assesses the quality of a translated text relative to the source text and assigns a numerical score. The SSA procedure similarly uses an LLM to evaluate the degree of semantic equivalence between the original and translated versions by comparing their meaning and content. Both methods generate quantitative scores accompanied by qualitative justifications and suggestions for improvement. Scores range from 0 to 100, with higher scores indicating greater translation quality. As there are currently no consensus guidelines or thresholds that define GEMBA-DA and SSA translation scores, the equivalence criteria were informed by recommendations for AI-translation scoring, where a score of 80 or above is considered indicative of good translation quality and a score of 100 represents a perfect translation [39]. Therefore, thresholds were set at ≥90 for the equivalence criteria. The final translation was adopted when the equivalence criteria were met. The procedure was complemented with input from topic experts and developers of the DCAPP instrument in the translation and back-translation workflow process. A free version of DeepL online and a paid version of GPT-4.0 (Plus) were used during the adaptation and translation process between April 1 and April 9, 2025.

The procedure began with an initial forward translation of the DCAPP items from English to Swedish using DeepL. The translated items were then manually reviewed and edited, if needed, by bilingual members of the research team with expertise in digital psychology to ensure the preservation of domain-specific terminology and contextual relevance. Subsequently, each translated item was evaluated using GPT-based metrics, including GEMBA-DA and SSA, which generated a score, a justification, and item-specific suggestions for improvement by comparing the original and translated versions.

Items with GEMBA-DA and SSA scores of 90 or above proceeded directly to back-translation. For items with GEMBA-DA or SSA scores below 90, an iterative revision process was undertaken using the feedback generated by ChatGPT in combination with researcher expertise. This process continued until item-level evaluations indicated high similarity between the source and translated versions, defined as GEMBA-DA and SSA scores of 90 or above. Three items in the Abilities subscale (items 7, 11, and 12) were retained despite not meeting the predefined equivalence criterion. Following expert review, these translations were judged to provide better contextual and conceptual equivalence than alternative wordings, despite receiving GEMBA-DA and SSA scores below the predefined threshold. Following satisfactory evaluation, the Swedish items were back-translated into English using DeepL to facilitate item-level comparison with the original version. Finally, the translated and back-translated versions were reviewed by an expert committee comprising members of the research team and DCAPP developers to identify and resolve any remaining discrepancies.

The item-level evaluation, including prompts, GEMBA-DA and SSA score exports, item feedback from ChatGPT per iteration, and all manual changes with their justifications, was documented and saved in a translation log. The prompts used and an example of an item translation log can be found in Multimedia Appendices 4 and 5. The final Swedish version used in the survey can be found in Multimedia Appendix 6.

### Statistical Analysis

All quantitative data were processed and analyzed using descriptive and inferential statistical procedures in IBM SPSS Statistics version 29.0. Item-level descriptive statistics were calculated for all items within the Knowledge and Abilities subscales to assess response distributions and psychometric performance. For each item, the mean, SD, skewness, and kurtosis were computed. The internal consistency of the instrument was assessed for the total score and subscales (Knowledge and Abilities) using Cronbach α, a metric that evaluates the extent to which items within a scale and subscale are interrelated and measure the same underlying construct [40,41]. In addition to α, the mean interitem correlation was computed for each subscale to further assess item homogeneity, with values between 0.15 and 0.50 considered acceptable, and higher values suggesting stronger internal coherence but also the potential for item redundancy [42].

Corrected item–total correlations (CITCs) were used to evaluate the relationship between each item and the total subscale score (excluding the item itself), with values above 0.30 considered acceptable indicators of adequate item discrimination [43]. Cronbach α if an item was deleted was examined to identify whether the removal of any item would substantially increase internal consistency, using a threshold difference of >0.2 as a potential indicator for item revision.

Responsiveness to change was assessed using paired-sample *t* tests comparing baseline and follow-up scores among respondents who attended the digital psychology course and completed both assessments (n=9). Knowledge and Abilities subscale scores were calculated by summing the item scores within each subscale. The total DCAPP score was calculated as the sum of all item scores. Statistical significance was set at *P*<.05 (2-tailed). The limited number of follow-up respondents among students not enrolled in the digital psychology course (n=3) precluded meaningful between-group comparisons. Consequently, responsiveness analyses were restricted to respondents who attended the course. Missing data were not imputed, and all analyses were conducted using available-case analysis.

### Ethical Considerations

The Swedish Ethical Review Authority determined that the project did not involve the processing of personal data as defined in Section 3 of the Swedish Ethical Review Act and that the study did not require formal ethical review (Dnr 2025-00064-01). All respondents had to provide informed consent digitally before accessing the survey (see Multimedia Appendix 7). Respondents could not proceed to the questionnaire without providing consent. Participation was voluntary, and no incentives were provided for participation in the survey. All analyses were conducted on anonymized data.

## Results

### AI-Assisted Translation of the DCAPP

The AI-assisted translation resulted in a Swedish version with a mean GEMBA-DA rating of 93 (SD 2.65, range 88‐97) and an SSA rating of 93 (SD 2.69, range 85‐97). Fifteen of 16 (94%) Abilities items and all 15 (100%) Knowledge items were manually modified after the initial DeepL translation to improve linguistic and contextual fit. Three of 16 (19%) Abilities items and 4 of 15 (27%) Knowledge items were further modified after receiving below-threshold GEMBA-DA or SSA ratings.

### Participant Characteristics

Characteristics of participants can be found in Table 1. The 6 participants who received training earlier in the master’s program had all attended a nonmandatory half-day lecture on videoconferencing psychotherapy in the same semester. Five of the 7 participants who reported a previous experience working with digital formats had served as online therapists in research studies.

**Table 1.** Participant characteristics and previous experience of digital psychology (N=24).

Characteristics	Value
Age (y), mean (SD)	25.67 (3.62)
Gender, n (%)	
Woman	19 (79)
Man	4 (17)
Nonbinary	0 (0)
Unsure	0 (0)
Prefer not to answer	1 (4)
Registered for the digital psychology course, n (%)	
Yes	13 (54)
No	11 (46)
Previous digital training at the master’s program, n (%)	
Yes	6 (25)
No	18 (75)
Previous digital training outside of the master’s program, n (%)	
Yes	1 (4)
No	23 (96)
Previous experience of working in digital formats, n (%)	
Yes	7 (29)
No	17 (71)

### Internal Consistency

The internal consistency of the total scale was excellent (Cronbach α=0.96, 95% CI 0.93-0.98). For the subscales, Cronbach α was 0.93 (95% CI 0.86-0.96) for Knowledge and 0.96 (95% CI 0.92-0.97) for Abilities. The mean inter-item correlation was 0.48 for the Knowledge items and 0.61 for the Abilities items. These values suggest that the items within each construct are internally consistent. The mean interitem correlation was slightly above the recommended range (>0.50) for the Abilities subscale, which may indicate potential item redundancy.

### Item-Level Performance (Knowledge Subscale)

Knowledge items in the DCAPP showed acceptable distributional properties. Means fell in the lower-to-mid range of the 1 to 5 Likert scale with modest dispersion, and skewness/kurtosis were small in magnitude for most items. Floor and ceiling percentages were generally low and consistent with adequate spread, although 5 items had floor percentages above 40%. Furthermore, items 13 and 14 displayed pronounced floor effects and marked positive skewness/kurtosis, indicating limited discrimination for these items. CITCs were positive and generally of acceptable magnitude above 0.30, and “α if deleted” indicated that all items contributed meaningfully to the composite Knowledge score. All statistics for the item-level performance of the Knowledge subscale can be found in Table 2.

**Table 2.** Item-level performance for the Knowledge subscale at baseline (N=24)^a^.

Item	Label (short)	Mean (SD)	Skew	Kurtosis	Floor, %	Ceiling, %	CITC^b^	α if deleted^c^
1	Opportunities and limitations	2.79 (0.78)	−0.21	−0.12	4.2	16.7	0.61	0.93
2	Diversity and cultural differences	2.04 (0.75)	0.60	0.83	20.8	4.2	0.54	0.93
3	Organizational and professional guidelines	1.63 (0.65)	0.54	−0.52	45.8^d^	5.3	0.78	0.92
4	Information governance, legal context, and data collection	1.63 (0.71)	0.71	−0.62	50.0^d^	12.5	0.75	0.92
5	Evidence base	2.38 (0.88)	−0.01	−0.57	16.7	8.3	0.63	0.93
6	Alliance and engagement	2.29 (0.91)	0.12	−0.69	20.8	8.3	0.56	0.93
7	Available digital tools	2.29 (0.91)	0.50	−0.27	16.7	12.5	0.74	0.92
8	Interventions for individuals	2.21 (0.83)	0.07	−0.61	20.8	4.2	0.62	0.93
9	Interventions for families, groups, or organizations	1.58 (0.78)	0.92	−0.64	58.3^d^	16.7	0.77	0.92
10	Communication processes	2.5 (0.89)	0.00	−0.53	12.5	12.5	0.56	0.93
11	Online teaching methods	2.33 (0.87)	0.13	−0.47	16.7	8.3	0.70	0.92
12	Supervision, leadership, and consultation	1.83 (0.70)	0.24	−0.81	33.3	16.7	0.84	0.92
13	Tools for clinical outcomes	1.63 (0.88)	1.28	0.87	58.3^d^	4.2	0.69	0.92
14	Clinical safety and risk management	1.46 (0.78)	1.96	4.02	66.7^d^	4.2	0.59	0.93
15	Own attitudes, skills, ethics, and practice	2.13 (0.80)	0.32	−0.10	20.8	4.2	0.63	0.93

a Items were rated from 1 to 5.

b CITC: corrected item–total correlation.

c “α if deleted” values refer to Cronbach α.

d The values above 40% indicate low discrimination.

### Item-Level Performance (Abilities Subscale)

Abilities items in the DCAPP also showed acceptable distributional properties. Item means fell in the lower-to-mid range of the scale, similar to the Knowledge items. SDs were somewhat higher than those for the Knowledge subscale, although skewness and kurtosis values remained within acceptable bounds for small samples. However, items 2 and 10 demonstrated pronounced positive skew and kurtosis, together with substantial floor effects (71% and 79% of responses at the lowest response category, respectively), indicating potential issues with item discrimination. Eight additional items (3, 4, 5, 6, 8, 9, 12, and 14) also showed moderate floor effects (≥40%). CITCs were consistently above the 0.30 benchmark, suggesting that each item contributed meaningfully to the total score. No item, if deleted, would have substantially increased Cronbach α, and the overall pattern supported the internal coherence and contribution of each item to the Abilities construct. All statistics for the item-level performance of the Abilities subscale can be found in Table 3.

**Table 3.** Item-level performance for the Abilities subscale at baseline (N=24)^a^.

Item	Label (short)	Mean (SD)	Skew	Kurtosis	Floor, %	Ceiling, %	CITC^b^	α if deleted^c^
1	Respect boundaries and therapeutic relationship	2.12 (1.12)	0.55	−1.02	37.5	16.7	0.73	0.95
2	Deliver culturally appropriate interventions	1.38 (0.71)	2.46	7.34	70.8^d^	4.2	0.68	0.95
3	Follow organizational and professional guidelines	1.58 (0.78)	0.92	−0.64	58.3^d^	16.7	0.65	0.96
4	Explain advantages and disadvantages	1.92 (1.06)	0.66	−1.00	50.0^d^	8.3	0.87	0.95
5	Conduct clinical risk and safety assessments	1.42 (0.65)	1.35	0.81	66.7^d^	8.3	0.79	0.95
6	Use an evidence-based approach	1.71 (0.75)	0.55	−0.95	45.8^d^	16.7	0.74	0.95
7	Introduce and engage clients	1.96 (0.95)	0.74	−0.24	37.5	8.3	0.83	0.95
8	Select and administer assessment tools	1.75 (0.90)	0.94	−0.01	50.0^d^	4.2	0.81	0.95
9	Deliver interventions to individuals	2.00 (1.02)	0.53	−0.95	41.7^d^	8.3	0.86	0.95
10	Deliver interventions to families, groups, or organizations	1.29 (0.62)	2.06	3.24	79.2^d^	8.3	0.72	0.95
11	Adapt communication style	2.08 (0.97)	0.75	−0.16	29.2	12.5	0.83	0.95
12	Deliver educational interventions	1.96 (1.08)	0.76	−0.72	45.8^d^	12.5	0.82	0.95
13	Conduct supervision, teamwork, and meetings	2.38 (1.28)	0.44	−1.08	33.3	4.2	0.53	0.96
14	Collect and manage outcome data	1.96 (1.00)	0.66	−0.65	41.7^d^	8.3	0.63	0.96
15	Reflect on own attitudes, skills, ethical considerations, and values	2.25 (1.19)	0.32	−1.44	37.5	20.8	0.87	0.95
16	Recognize limitations in skills	2.21 (0.98)	0.16	−1.04	29.2	8.3	0.80	0.95

a Items were rated from 1 to 5.

b CITC: corrected item–total correlation.

c “α if deleted” values refer to Cronbach α.

d The values above 40% indicate low discrimination.

### Responsiveness to Change

There were significant changes in both subscales and the total score for the group (N=9) that attended the digital psychology course and completed baseline and follow-up measurements (Table 4). Given the small sample size, these results should be interpreted with caution and considered preliminary.

**Table 4.** Change in DCAPP (Digital Competencies for Applied Psychological Practitioners) scores from baseline to follow-up among students attending the digital psychology course (N=9).

Scale	Pre, mean (SD)	Post, mean (SD)	Mean difference (SD)	95% CI	*t* test (*df*)	*P* value	*d*
Knowledge	29.33 (10.27)	51.44 (3.24)	−22.11 (9.68)	−29.55 to −14.67	−6.86 (8)	<.001	2.9
Abilities	26.00 (10.28)	51.44 (8.50)	−25.44 (10.49)	−33.51 to −17.38	−7.28 (8)	<.001	2.7
Total	55.33 (19.71)	102.89 (11.19)	−47.56 (19.33)	−62.41 to −32.70	−7.38 (8)	<.001	3.0

## Discussion

### Principal Findings

The aim of this study was to test an AI-assisted translation and adaptation procedure for the DCAPP scale in a Swedish context and to provide an initial psychometric evaluation of the translated instrument. The findings indicate that AI-assisted adaptation can be a feasible approach for the translation of competency measures, resulting in high translation quality and semantic similarity. The Swedish DCAPP demonstrated promising psychometric properties, including high internal consistency and acceptable item-level performance. Preliminary evidence of responsiveness to change was also observed among students enrolled in a digital psychology course, suggesting that the instrument may be capable of detecting changes in self-reported digital competencies following educational interventions.

### Implications for Training and Practice

The overall scores on the DCAPP were low at baseline, suggesting that students were not familiar with the topic of digital clinical psychology. Because 92% (22/24) of respondents stated a need for the knowledge and abilities to carry out digital clinical work, this confirms the perceived lack of digital competencies and lack of access to training for clinical psychology students in academic settings and among health care professionals in clinical settings [26,44]. The shortage of training could be due to difficulties in adapting the curriculum in higher education programs at the same pace as real-world settings, where the COVID-19 pandemic has increased the use of digital tools among health care professionals in mental health care practice [4,45]. The findings may also reflect differences in training programs and educational settings across national contexts. When piloting the competency framework with diploma-level trained CBT therapists from UK Talking Therapies services, feedback indicated that the competencies should be divided into “Core” (applicable to any psychological professional at diploma or doctoral level) and “Advanced” (typically only necessary on a doctoral course) [30]. Differences between UK and Swedish clinical psychology curricula may reflect this distinction and may explain the lower baseline scores observed in the present study. The unfamiliarity with digital ways of working among Swedish psychology master’s students could contribute to a lack of preparedness for digital clinical work, a challenge reported among health care professionals in general [46]. It may also result in missed opportunities for discussion and experience of the pros and cons of transitioning from in-person to digital formats [15-17,47]. Not adequately preparing psychology students for the effects of digitalization in mental health care systems, including the consequences for psychologists within these systems, could leave them unprepared for a transformed role as clinical psychologists [22,48]. Such preparation should also include developing competencies related to AI through educational activities and curriculum integration [49,50].

Respondents reported the lowest levels of knowledge and abilities related to digital interventions for families, groups, and organizations, as well as clinical management and safety issues in digital provision. These items relate to a broader scope of digital clinical psychology and are not limited to the provision of digital mental health interventions for individuals, as the competency framework underpinning the DCAPP was developed to reflect a wide range of competencies typically included in professional psychology training, including clinical and counseling psychology. However, to be disseminated more broadly, the instrument should be applicable to a variety of educational and training settings. The content and structure of the digital psychology course in this study were based on a Swedish course book on the topic and not on the UK competency framework [51]. Even so, the DCAPP showed preliminary responsiveness to change. A factor influencing its generalizability could be that the adaptation process of the instrument included discussions in the research group regarding the wider applicability of wording used in the scale. This included removing contextually specific terms to create a Swedish version and considering how a back-translated version of the scale could be used as a template for adaptation to other national contexts. An updated English version of the DCAPP was therefore created after the study, incorporating learnings from the adaptation of the Swedish version [31].

The lower scores on the Abilities subscale and greater variability in responses (higher SDs) are most likely the result of a lack of training in digital formats and practically applicable digital competencies. This is not surprising, as psychology master’s students in Sweden generally have fewer practice-oriented educational activities than activities focusing on knowledge acquisition [52]. Students at Linköping University start their clinical training in year 4, which includes the assessment and treatment of clients with mental health problems. The question of how and when to provide digital clinical competencies throughout the master’s program is therefore of relevance. Usually, training in psychotherapy is preceded by learning about therapeutic models of delivery. If the same is to apply for digital clinical competencies, a complete competency acquisition should take into consideration that the actual use of digital formats should be preceded by a course focusing on knowledge acquisition or be delivered in an integrated fashion. One example of an integrated format is the stand-alone course at Uppsala University in Sweden on internet-based CBT, where students shortly after and in parallel with acquiring knowledge also treat patients under supervision as part of their training [53].

The limited competence in delivering culturally appropriate interventions identified in this study is consistent with findings from previous surveys [44,54]. This is an important topic to address in educational activities and in the development of clinical interventions for mental health problems, as there is limited research on the use, implementation, and role of psychologists in the delivery of digital mental health interventions for culturally diverse, underserved, and marginalized groups [55]. However, there is potential for AI to help improve the training of future therapists by including multicultural competencies applicable to digital health interventions [56] and assisting with the adaptation of therapeutic content [57].

AI-informed procedures can already generate translations of similar quality to human translations [35]. Using such procedures can save time and resources for researchers and developers, facilitating data collection in new contexts and across borders. However, the risks need to be addressed as well, as an automated AI process can be restricted by inherent biases in generative AI systems, and the subtleties of cultural and linguistic differences may not be fully captured [58]. Standard procedures for translation, cultural adaptation, and linguistic validation, using well-defined and rigorous processes, are still the gold standard [32], and AI translations are currently more dependent on the vocabulary and cultural similarity between the source and the target language [35]. If using AI-assisted procedures, manual review and modification by bilingual researchers and clinicians with relevant expertise and contextual knowledge remain necessary. GEMBA-DA and SSA ratings may appear objective, but there is a need to develop and define agreed-upon thresholds and compare them with gold-standard translation procedures to confirm the quality and semantic similarity of translations assessed using these metrics.

### Limitations

This study represents an initial step in validating DCAPP and the adapted Swedish version of the instrument. However, several limitations should be noted. The small sample size and lack of a power analysis restrict the conclusions that can be drawn from statistical analyses and psychometric estimates. Reliability coefficients derived from small samples are associated with greater uncertainty, and the responsiveness analyses were based on a limited number of participants, reducing statistical power. Furthermore, the sample size precluded the examination of the scale’s factor structure (eg, through exploratory or confirmatory analyses), limiting conclusions regarding its dimensionality. Consequently, the psychometric findings should be considered preliminary pending replication and more comprehensive validation in larger samples.

Several items, primarily within the Abilities subscale, demonstrated pronounced floor effects and skewed distributions, which may limit their sensitivity to change and their ability to discriminate among respondents. While this likely reflects the limited practical experience of psychology students with digital service delivery, it is also possible that certain items are less suitable for respondents with limited clinical exposure. The observed response patterns could reflect differences between the target population (students) and the original intended users of the instrument (practitioners) rather than shortcomings in the items themselves, indicating that certain items could benefit from further refinement to improve their alignment with the intended construct and diverse target populations. Although overall reliability was high, future validation studies involving both students and practicing psychologists are needed to determine whether the observed floor effects primarily reflect characteristics of the sample, the suitability of specific items for different populations, or limitations of individual items.

We used convenience sampling, and conclusions were drawn from a single university and a specific academic context, which may limit the external validity of the findings. The results may not generalize to students in other psychology master’s programs or to practicing psychologists. Broader sampling across training levels and institutions is needed to strengthen the instrument’s applicability. We did not use established guidelines, such as those developed by the International Society for Pharmacoeconomics and Outcome Research [32], in our translation process since the purpose of the study was to examine the use of an AI-assisted translation process. As no direct comparison with gold-standard translation procedures was conducted, it remains unclear how the translation quality compares with that achieved using established translation methods.

### Conclusions

The increasing digitalization of psychological services is transforming the competencies required for current and future psychological practitioners. Reliable instruments are therefore needed to support the assessment, development, and evaluation of digital competencies in both educational and clinical settings. This study provides preliminary support for the AI-adapted Swedish version of the DCAPP and suggests that it may be useful for evaluating digital competency development among psychology students. Beyond the instrument itself, the findings indicate that AI-assisted translation and adaptation procedures may represent a feasible approach for accelerating the cross-contextual adaptation of competency measures and other questionnaires. However, although the present findings provide preliminary support for the Swedish version of the DCAPP, further validation is warranted. Future studies should include larger and more heterogeneous samples, enabling exploratory and confirmatory factor analyses as well as more comprehensive assessments of construct validity, test-retest reliability, and responsiveness to change. Establishing these psychometric properties will be important for determining the scale’s suitability for educational, clinical, and research applications. In addition, the use of AI-assisted procedures for questionnaire translation and adaptation should include comparisons with gold-standard procedures to confirm their potential benefits.

## Supplementary material

10.2196/92332 Multimedia Appendix 1 Baseline questions.

10.2196/92332 Multimedia Appendix 2 Follow-up questions.

10.2196/92332 Multimedia Appendix 3 Description of the online survey platform (Iterapi).

10.2196/92332 Multimedia Appendix 4 GPT Estimation Metric-Based Assessment–Direct Assessment and custom Semantic Similarity Assessment prompts.

10.2196/92332 Multimedia Appendix 5 Translation log Digital Competencies for Applied Psychological Practitioners item 4 Knowledge.

10.2196/92332 Multimedia Appendix 6 Digital Competencies for Applied Psychological Practitioners—Swedish edition.

10.2196/92332 Multimedia Appendix 7 Informed consent.

10.2196/92332 Checklist 1 CHERRIES checklist.

## Abbreviations

CBT: cognitive behavioral therapy
CHERRIES: Checklist for Reporting Results of Internet E-Surveys
CITC: corrected item–total correlation
DCAPP: Digital Competencies for Applied Psychological Practitioners
ECTS: European Credit Transfer and Accumulation System
GEMBA-DA: GPT Estimation Metric Based Assessment–Direct Assessment
LLM: large language model
SSA: Semantic Similarity Assessment

## References

[1] Zale A, Lasecke M, Baeza-Hernandez K (2021). Technology and psychotherapeutic interventions: bibliometric analysis of the past four decades. Internet Interv.

[2] Vernmark K, Buhrman M, Carlbring P, Hedman-Lagerlöf E, Kaldo V, Andersson G (2024). From research to routine care: a historical review of internet-based cognitive behavioral therapy for adult mental health problems in Sweden. Digit Health.

[3] Wind TR, Rijkeboer M, Andersson G, Riper H (2020). The COVID-19 pandemic: the “black swan” for mental health care and a turning point for e-health. Internet Interv.

[4] Feijt M, de Kort Y, Westerink J, Bierbooms J, Bongers I, IJsselsteijn W (2022). Integrating technology in mental healthcare practice: a repeated cross-sectional survey study on professionals’ adoption of Digital Mental Health before and during COVID-19. Front Psychiatry.

[5] Li H, Zhang R, Lee YC, Kraut RE, Mohr DC (2023). Systematic review and meta-analysis of AI-based conversational agents for promoting mental health and well-being. NPJ Digit Med.

[6] Cross S, Bell I, Nicholas J (2024). Use of AI in mental health care: community and mental health professionals survey. JMIR Ment Health.

[7] de Graaff AM, Habashneh R, Fanatseh S (2025). Evaluation of a guided chatbot intervention for young people in Jordan: feasibility randomized controlled trial. JMIR Ment Health.

[8] Leuchtenberg S, Gromer D, Käthner I (2023). Videoconferencing versus face‐to‐face psychotherapy: insights from patients and psychotherapists about comparability of therapeutic alliance, empathy and treatment characteristics. Couns Psychother Res.

[9] Hedman-Lagerlöf E, Carlbring P, Svärdman F, Riper H, Cuijpers P, Andersson G (2023). Therapist-supported Internet-based cognitive behaviour therapy yields similar effects as face-to-face therapy for psychiatric and somatic disorders: an updated systematic review and meta-analysis. World Psychiatry.

[10] Koelen JA, Vonk A, Klein A (2022). Man vs. machine: a meta-analysis on the added value of human support in text-based internet treatments (“e-therapy”) for mental disorders. Clin Psychol Rev.

[11] Karyotaki E, Efthimiou O, Miguel C (2021). Internet-based cognitive behavioral therapy for depression: a systematic review and individual patient data network meta-analysis. JAMA Psychiatry.

[12] Domhardt M, Geßlein H, von Rezori RE, Baumeister H (2019). Internet- and mobile-based interventions for anxiety disorders: a meta-analytic review of intervention components. Depress Anxiety.

[13] Malouin-Lachance A, Capolupo J, Laplante C, Hudon A (2025). Does the digital therapeutic alliance exist? Integrative review. JMIR Ment Health.

[14] Vernmark K, Hesser H, Topooco N (2019). Working alliance as a predictor of change in depression during blended cognitive behaviour therapy. Cogn Behav Ther.

[15] Erlandsson A, Forsström D, Rozental A, Werbart A (2022). Accessibility at what price? Therapists’ experiences of remote psychotherapy with children and adolescents during the COVID-19 pandemic. J Infant Child Adolesc Psychother.

[16] De Witte NAJ, Carlbring P, Etzelmueller A (2021). Online consultations in mental healthcare during the COVID-19 outbreak: an international survey study on professionals’ motivations and perceived barriers. Internet Interv.

[17] Weineland S, Ribbegårdh R, Kivi M (2020). Transitioning from face-to-face treatment to iCBT for youths in primary care—therapists’ attitudes and experiences. Internet Interv.

[18] Vernmark K, Norlin M, Andersson G (2026). Clinicians’ attitudes towards videoconferencing cognitive behavioural therapy: an exploratory survey. Cogn Behav Ther.

[19] Jarva E, Oikarinen A, Andersson J, Tomietto M, Kääriäinen M, Mikkonen K (2023). Healthcare professionals’ digital health competence and its core factors; development and psychometric testing of two instruments. Int J Med Inform.

[20] González-Robles A, Miguel C, Richards D, Duffy D, Enrique Á (2024). A scoping review of therapist behaviors in guided digital mental health interventions. Internet Interv.

[21] (2025). Standards for the accreditation of doctoral programmes in clinical psychology. https://cms.bps.org.uk/sites/default/files/2022-07/Clinical%20Psychology%20-%20Standards%20for%20Accreditation.pdf.

[22] Gambirasio M, Ivaldi S, Scaratti G (2024). How platformisation transforms the psychological profession: reflections from a proposal of indicators for the classification of psychological platforms. Community Applied Soc Psy.

[23] Carl JR, Jones DJ, Lindhiem OJ (2022). Regulating digital therapeutics for mental health: opportunities, challenges, and the essential role of psychologists. Br J Clin Psychol.

[24] Van Kessel K (2021). Clinical psychologists’ attitudes towards using technology in therapy: a survey. J N Z Coll Clin Psychol.

[25] Ruzek JI, Sadeh-Sharvit S, Bunge EL (2024). Training the psychologist of the future in the use of digital mental health technologies. Prof Psychol: Res Pr.

[26] Pote H, Rees A, Holloway-Biddle C, Griffith E (2021). Workforce challenges in digital health implementation: how are clinical psychology training programmes developing digital competences?. Digit Health.

[27] Pote H, Moulton-Perkins A, Rides G (2020). Competence framework for digital clinical practice: psychological practitioners. https://digitalhealthskills.com/digitalcompetencies.

[28] Pote H, Moulton-Perkins A, Rides G, Pote H, Moulton-Perkins A, Campbell A (2025). Psychological Digital Practice: The Basics and Beyond.

[29] Moulton-Perkins A, Pote H, Latchford G (2022). Developing competencies for digital clinical practice: elearning. https://digitalhealthskills.com/elearning/.

[30] Pote H, Moulton-Perkins A, Rides G (2020). Digital competencies for psychological practitioners in IAPT services. a report for IAPT london. https://ora.ox.ac.uk/objects/uuid:efd776f9-d538-4558-a72f-d1d3e155e74c.

[31] Pote H, Moulton-Perkins A, Rides G (2024). Development of the Digital Competencies for Applied Psychological Practitioners (DCAPP) scale [OSF project]. Open Science Framework.

[32] McKown S, Acquadro C, Anfray C (2020). Good practices for the translation, cultural adaptation, and linguistic validation of clinician-reported outcome, observer-reported outcome, and performance outcome measures. J Patient Rep Outcomes.

[33] Haavisto O, Welsch R (2024). Questionnaires for everyone: streamlining cross-cultural questionnaire adaptation with GPT-based translation quality evaluation. arXiv.

[34] Kocmi T, Federmann C, Nurminen M, Brenner J, Koponen M (2023). Proceedings of the 24th Annual Conference of the European Association for Machine Translation.

[35] Kunst JR, Bierwiaczonek K (2023). Utilizing AI questionnaire translations in cross-cultural and intercultural research: insights and recommendations. Int J Intercult Relat.

[36] (2023). Exploring GEMBA: a new LLM-based metric for translation quality assessment. Medium.

[37] Eysenbach G (2004). Improving the quality of web surveys: the Checklist for Reporting Results of Internet E-Surveys (CHERRIES). J Med Internet Res.

[38] Vlaescu G, Alasjö A, Miloff A, Carlbring P, Andersson G (2016). Features and functionality of the Iterapi platform for internet-based psychological treatment. Internet Interv.

[39] Krukowski I (2025). Scoring translation quality. Lokalise.

[40] Cronbach LJ (1951). Coefficient alpha and the internal structure of tests. Psychometrika.

[41] Tavakol M, Dennick R (2011). Making sense of Cronbach’s alpha. Int J Med Educ.

[42] Clark LA, Watson D (1995). Constructing validity: basic issues in objective scale development. Psychol Assess.

[43] Ferketich S (1991). Focus on psychometrics. Aspects of item analysis. Res Nurs Health.

[44] Dobson R, Variava R, Douglas M, Reynolds LM (2022). Digital competency of psychologists in Aotearoa New Zealand: a cross-sectional survey. Front Digit Health.

[45] Ellis LA, Meulenbroeks I, Churruca K (2021). The application of e-mental health in response to COVID-19: scoping review and bibliometric analysis. JMIR Ment Health.

[46] Van Daele T, Mathiasen K, Carlbring P (2022). Online consultations in mental healthcare: modelling determinants of use and experience based on an international survey study at the onset of the pandemic. Internet Interv.

[47] Ibragimov K, Palma M, Keane G (2022). Shifting to tele-mental health in humanitarian and crisis settings: an evaluation of Médecins Sans Frontières experience during the COVID-19 pandemic. Confl Health.

[48] Stein OA, Prost A (2024). Exploring the societal implications of digital mental health technologies: a critical review. SSM - Ment Heal.

[49] Åhs F, Mozelius P, Espvall M (2022). Preparing psychologists and social workers for the daily use of AI. Int Conf AI Res.

[50] Gado S, Kempen R, Lingelbach K, Bipp T (2022). Artificial intelligence in psychology: how can we enable psychology students to accept and use artificial intelligence?. Psychol Learn Teach.

[51] Edbacken J, Vernmark K (2021). Digital Psykologi: Forskning Och Klinisk Tillämpning [Book in Swedish].

[52] (2025). Kartläggning av klinisk färdighetsträning för psykologstudenter–beroende och primärvård [Report in Swedish]. https://www.kliniskapsykologer.se/kartlaggning-av-klinisk-fardighetstraning-for-psykologstudenter/.

[53] (2026). Digital psykologi i teori och praktik [Article in Swedish]. Uppsala universitet.

[54] Vernmark K, Geranmayeh A, Topooco N, Andersson G, Shahnavaz S (2026). Understanding psychologists’ usage, knowledge, and attitudes toward digital mental health solutions for refugees and migrants: exploratory cross-sectional survey in Sweden. JMIR Hum Factors.

[55] Whitehead L, Talevski J, Fatehi F, Beauchamp A (2023). Barriers to and facilitators of digital health among culturally and linguistically diverse populations: qualitative systematic review. J Med Internet Res.

[56] Shafran R, Bond L, Carlbring P (2026). From innovation to implementation: artificial intelligence in cognitive behaviour therapy training and supervision. Behav Res Ther.

[57] Demetry Y, Carlbring P, Andersson G (2026). Cultural relevance and acceptability of cognitive behavioral therapy techniques adapted by AI or a human psychologist: experimental study. JMIR Form Res.

[58] Karpouzis K (2024). Plato’s shadows in the digital cave: controlling cultural bias in generative AI. Electronics.

