# Supplementary material for: AI-Assisted Adaptation of the Swedish Version of the Digital Competencies for Applied Psychological Practitioners Scale: Preliminary Psychometric Evaluation
Source: JMIR Form Res. 2026 Sep 8;10:e92332. doi: 10.2196/92332 (PMC13552648; doi:10.2196/92332)
Supplement: Supplementary file 1 [file formative-v10-e92332-s001.docx]

Demografi + övrigt

**Kommer du att delta i kursen *Digital psykologi - forskning och klinisk tillämpning (735A40)* med start den 25e april?**

Ja/Nej

**Vilken ålder är du?**

Nummer

**Vilket kön har du? Med kön menar vi könsidentitet, alltså det kön du själv känner dig som**

- Kvinna
- Man
- Ickebinär
- Annat alternativ
- Osäker
- Vill ej svara

**Har du fram till nu deltagit vid något utbildningsmoment om digital psykologi på psykologprogrammet vid Linköpings Universitet?**

Ja/nej

Om ja,

Beskriv det/de utbildningsmoment du deltagit på

Fritext

**Har du gått någon tidigare utbildning i digital psykologi, utöver det som du deltagit i via psykologprogrammet vid Linköpings Universitet?**

Ja/Nej

Om ja,

Beskriv den utbildning du deltagit på

Fritext

**Har du någon tidigare erfarenhet av psykologiskt arbete i digitalt format?**

Ja/Nej

Om ja,

Beskriv din erfarenhet av digitalt psykologarbete

Fritext

**Anser du att psykologer behöver digitala kunskaper och färdigheter för att kunna bedriva sitt kliniska arbete?**

Ja/Nej

Utveckla gärna dina tankar om psykologers eventuella behov, eller avsaknad av behov, av digitala kunskaper och färdigheter

Fritext

--

**Tack för ditt deltagande i denna enkät!**

Om cirka åtta veckor är det dags att fylla i enkäten igen. Det är viktigt att vi får in dina svar även vid nästa tillfälle. När det är dags kommer du att få ett mail med en länk till enkäten skickat till den mailadress du angav när du registrerade dig för studien.
