# Supplementary material for: AI-Assisted Adaptation of the Swedish Version of the Digital Competencies for Applied Psychological Practitioners Scale: Preliminary Psychometric Evaluation
Source: JMIR Form Res. 2026 Sep 8;10:e92332. doi: 10.2196/92332 (PMC13552648; doi:10.2196/92332)
Supplement: Supplementary file 2 [file formative-v10-e92332-s002.docx]

Text till andra utskicket

Nu är det dags att för den andra och avslutande delen av enkätundersökningen. För att kunna utvärdera instrumentet och dess nytta så vill vi att du svarar på samma frågor ytterligare en gång, samt ger din feedback på frågorna och dess utformning.

Vi är tacksamma för din input!

--

Har du sedan du fyllde i enkäten vid första tillfället deltagit i någon utbildning eller färdighetsträning i digital klinisk psykologi (utöver den valbara kursen i Digital psykologi vid Psykologprogrammet)?

Ja/Nej

Om ja

Beskriv vilken typ av utbildning

-

Nedan följer alla frågor du svarat på i denna enkät. Vi vill gärna få din feedback på hur lätt eller svårt det har varit att förstå innebörden i de enskilda frågorna. I fritextfälten vid varje enskild fråga kan du ge dina synpunkter på formulering, begriplighet, relevans, eller annan input som du anser vara relevant. Ge gärna konkreta förslag på förbättringar av frågan!

Frågor om Kunskap

1. En förståelse för möjligheter och begränsningar med digitala arbetssätt
2. En förståelse för mångfald och kulturella skillnaders roll i användningen av digitala arbetssätt
3. En förståelse för klinisk styrning, samt organisatoriska och professionella riktlinjer, relaterade till digitala arbetssätt
4. En förståelse för informationsstyrning och de juridiska förutsättningarna för insamling och lagring av klientdata vid användning av digitala metoder
5. En förståelse av evidensbasen för digitala arbetssätt
6. En förståelse för frågor kring klinisk allians och engagemang vid digitalt klientarbete
7. En förståelse för de psykologiska verktyg som finns tillgängliga att administrera digitalt
8. En förståelse för digitala interventioner riktade till individer
9. En förståelse för digitala interventioner riktade till familjer, grupper eller organisationer
10. En förståelse för hur digitala arbetssätt påverkar kommunikationsprocesser (exempelvis turtagning och användning av icke-verbal information)
11. En förståelse av för- och nackdelar med onlinebaserade undervisningsmetoder och digitala undervisningsprogram
12. En förståelse för hur handledning, ledarskap, och konsultationer behöver anpassas vid digitala arbetssätt
13. Kunskap om digitala verktyg för att mäta kliniska utfall, monitorera, samt för utvärdering av tjänster och forskning
14. En förståelse för klinisk säkerhet och riskhantering i samband med digitalt arbete på distans
15. En förståelse för egna attityder, färdigheter, etiska principer och värderingar i förhållande till digitala arbetssätt

(Fritext vid varje fråga – inte obligatoriska svar)

Frågor om Förmågor

1. Förmåga att respektera gränser och utveckla en positiv terapeutisk relation online
2. Förmåga att förmedla kulturellt anpassade psykologiska insatser i digitalt format, inklusive genom tolkanvändning
3. Förmåga att följa organisatoriska och professionella riktlinjer avseende klinisk styrning och informationsstyrning för digitala arbetssätt
4. Förmåga att förklara för- och nackdelar med digitala förmedlingssätt för klienter och få deras informerade samtycke
5. Förmåga att genomföra korrekta kliniska risk- och säkerhetsbedömningar i användningen av digitala arbetssätt
6. Förmåga att använda ett evidensbaserat förhållningssätt till att kritiskt utvärdera digitala verktyg och interventioner för användning kliniskt och i forskning
7. Förmåga att introducera och engagera klienter till att använda digitala format
8. Förmåga att välja och administrera digitala psykologiska bedömningsverktyg utifrån klientens behov
9. Förmåga att förmedla digitala psykologiska interventioner riktade till individer
10. Förmåga att förmedla digitala psykologiska interventioner riktade till familjer, grupper eller organisationer
11. Förmåga att anpassa sin kommunikationsstil till klientens behov och det digitala format som används
12. Förmåga att förmedla utbildande insatser i det digitala arbetet med klienter och professionella (exempelvis psykoedukation och distansundervisning)
13. Förmåga att genomföra handledning, arbeta i team och delta i digitala möten på distans
14. Förmåga att digitalt samla in och hantera utfallsdata i kliniskt och forskningssyfte
15. Förmåga att reflektera över sina egna attityder, färdigheter, etiska överväganden och värderingar i förhållande till digitala arbetssätt
16. Förmåga att upptäcka begränsningarna i sin egen kompetens inom digitala arbetssätt

(Fritext vid varje fråga – inte obligatoriska svar)

Har det varit möjligt för dig att särskilja dina *kunskaper* och *förmågor* när du svarat på frågorna?

Ja/Nej

Om Nej

Förklara på vilket sätt det varit svårt att särskilja kunskaper och förmågor

Du har använt skalan med nedanstående skalsteg för att svara på frågorna. Är det något i denna skala och dess beskrivning som enligt dig behöver förändras eller förtydligas för att det ska vara lättare att svara på frågorna i enkäten.

1. Nybörjare: inga digitala kunskaper eller färdigheter
2. Mycket begränsad kompetens: behöver fortsatt en stor mängd kunskap och träning
3. Viss kompetens: behöver till viss del fortsatt träning och kunskap
4. Mestadels kompetent: behöver eventuellt klargöranden inom vissa områden
5. Expert: behöver ingen ytterligare kunskap eller träning

Fritext

Har du någon ytterligare feedback på enkäten som du vill skicka med?

Fritext

Stort tack för din medverkan!
