# Supplementary material for: AI-Assisted Adaptation of the Swedish Version of the Digital Competencies for Applied Psychological Practitioners Scale: Preliminary Psychometric Evaluation
Source: JMIR Form Res. 2026 Sep 8;10:e92332. doi: 10.2196/92332 (PMC13552648; doi:10.2196/92332)
Supplement: Supplementary file 3 [file formative-v10-e92332-s003.docx]

The Iterapi platform used for this study is a secure and widely used solution for providing online surveys, questionnaires, and internet-based interventions [50]. The servers are physically located at Linköping University, Sweden, in a locked computer room accessible only to authorized personnel. A responsive website compatible with web browsers on mobile phones, tablets, and computers was set up to provide information about the study, including the possibility to register and provide informed consent. All communication between servers and users was encrypted, and the survey utilized its own dedicated database, configuration files, database passwords, and encryption keys. Survey data was stored in an encrypted format within the database, using advanced algorithms and secret keys to prevent unauthorized linkage between stored data and individual users. The servers, where the data was stored, were continuously monitored and updated following well-defined routines by the local security team at Linköping University. Access to user data was restricted to authorized members of the research team.

The website and survey were open-access, meaning they were not password-protected and could be accessed by anyone with the survey link. To maintain anonymity, no client IP addresses were collected or stored. A cookie was used to prevent duplicate responses from the same browser and to recall partially saved answers if respondents completed the survey in multiple sessions. Respondents were able to revise their answers while completing the survey by using the Back and Forward buttons. Once the survey was submitted, answers could no longer be modified. There was no function for the participants to download their survey answers during or after the completion of the survey.

The baseline survey consisted of four webpages: informed consent, the DCAPP, participant characteristics, and a final page with repeated key information about the study. The follow-up survey consisted of five webpages: an introduction, the DCAPP, feedback on DCAPP-items, feedback on subscale differentiation and the Likert-scale, and a final page with a short appreciation for participating in the survey.
