# Supplementary material for: AI-Assisted Adaptation of the Swedish Version of the Digital Competencies for Applied Psychological Practitioners Scale: Preliminary Psychometric Evaluation
Source: JMIR Form Res. 2026 Sep 8;10:e92332. doi: 10.2196/92332 (PMC13552648; doi:10.2196/92332)
Supplement: Supplementary file 4 [file formative-v10-e92332-s004.docx]

GEMBA-DA and SSA prompts in ChatGPT4o

GEMBA-DA

You are a professional translation evaluator. Evaluate the translation quality of the following source and target texts. Use the GEMBA-DA framework to rate the adequacy and fluency of the translation on a scale from 0 to 100, where 100 indicates perfect translation quality. Focus on semantic preservation, grammatical accuracy, and naturalness. Do not use a human reference translation — evaluate based on your internal knowledge.

Source (Original Language): " {original_text} "

Translation (Target Language): "{translated_text}"

Return your answer as JSON in the following format:

{

"score": 0-100,

"reasoning": "Brief explanation of the score focusing on accuracy and fluency",

"suggestions": "Suggestions for improving the translation if applicable"

}

SSL

Assess the semantic similarity of the following texts in English and Swedish on a scale from 0 (no semantic similarity at all) to 100 (perfect semantic similarity). Justify the score. Provide a single paragraph suggesting changes to the Swedish version (i.e. word or expression replacements) to improve the score.

Source (Original Language): " {original_text} "

Translation (Target Language): "{translated_text}"

Respond with JSON only in the following format:

{

"score": 0-100,

"reasoning": "...",

"suggestion": "..."

}
