# Supplementary material for: AI-Assisted Adaptation of the Swedish Version of the Digital Competencies for Applied Psychological Practitioners Scale: Preliminary Psychometric Evaluation
Source: JMIR Form Res. 2026 Sep 8;10:e92332. doi: 10.2196/92332 (PMC13552648; doi:10.2196/92332)
Supplement: Supplementary file 5 [file formative-v10-e92332-s005.docx]

DCAPP Item 4 – Knowledge competencies

*An understanding of* ***information governance and the legal context*** *for collecting and storing client data using digital methods*

En förståelse för **informationsstyrning och de juridiska förutsättningarna** för insamling och lagring av klientdata vid användning av digitala metoder

--

Translation process – DeepL (Free version, 250401)

*Original (english)*

An understanding of **information governance and the legal context** for collecting and storing client data using digital methods

*Translation (Swedish)*

Förståelse för informationsstyrning och det juridiska sammanhanget för insamling och lagring av kunddata med hjälp av digitala metoder

*Manual changes*

”Informationsstyrning” could be replaced by ”informationshantering” or ”datahantering” to better adress the handling of data. ”Kunddata” replaced by ”klientdata”. ”Sammanhanget” replaced by ”förutsättningar” to increase clarity in the Swedish version.

*Updated item*

En förståelse för informationsstyrning och de juridiska förutsättningarna för insamling och lagring av klientdata vid användning av digitala metoder

*Backtranslation (to English)*

An understanding of information governance and the legal requirements for collecting and storing client data when using digital methods

Scoring – ChatGPT 4o (Paid version, 250401)

*GEMBA-DA*

{

"score": 97,

"reasoning": "The translation is highly accurate and grammatically sound. 'Informationsstyrning' is an appropriate term for 'information governance' in a professional or regulatory context. 'De juridiska förutsättningarna för insamling och lagring av klientdata' is a precise and natural rendering of 'the legal context for collecting and storing client data'. The phrase 'vid användning av digitala metoder' clearly reflects the original 'using digital methods'. Overall, the translation is fluent and captures the full meaning of the source.",

"suggestions": "No major changes are needed. If aiming for slightly more formal tone, you might consider 'i samband med användning av digitala metoder', though the current version is fully acceptable."

}

*SSA*

{

"score": 96,

"reasoning": "The translation closely matches the semantic content of the original sentence. 'Informationsstyrning' accurately reflects 'information governance', and the structure and phrasing of 'de juridiska förutsättningarna för insamling och lagring av klientdata' aligns well with the English source. The expression 'vid användning av digitala metoder' effectively communicates 'using digital methods', though the nuance of 'legal context' might be slightly broader than 'förutsättningar', which leans toward 'conditions' rather than legal framework.",

"suggestion": "For even tighter alignment, consider using 'juridiska ramar' or 'juridisk kontext' instead of 'juridiska förutsättningar', e.g. '...och den juridiska kontexten för insamling och lagring...'. This would more directly mirror the original phrasing."

}

Reflections

”Informationsstyrning” could be a word that the respondent have a hard time defining for themselves, and ”informationshantering” or ”datahantering” could be more useful. However, we have kept the original phrasing to stay more close to the original version. The word ”context” was translated to ”förutsättningar”, since it was considered a more understandable term than the word ”kontext” in this sentence.
