# Supplementary material for: AI-Assisted Adaptation of the Swedish Version of the Digital Competencies for Applied Psychological Practitioners Scale: Preliminary Psychometric Evaluation
Source: JMIR Form Res. 2026 Sep 8;10:e92332. doi: 10.2196/92332 (PMC13552648; doi:10.2196/92332)
Supplement: Supplementary file 6 [file formative-v10-e92332-s006.docx]

Digital Competencies for Applied Psychological Practitioners – Swedish Edition (DCAPP-SE)

Syftet med den här enkäten är en självskattning av dina kompetenser i digital klinisk psykologi. Frågorna är baserade på det kompetensramverk som utvecklats av British Psychological Society (Clinical Division) och har översatts och anpassats till en svensk kontext. Skatta dig själv på varje fråga utifrån den femgradiga skalan. Beskrivningarna av varje skalsteg är avsedda att användas som stöd vid skattningarna.

| 1 | 2 | 3 | 4 | 5 |
| --- | --- | --- | --- | --- |
| Nybörjare | Mycket begränsad  kompetens | Viss kompetens | Mestadels kompetent | Expert |
| inga digitala kunskaper eller färdigheter | behöver fortsatt en stor mängd kunskap och  träning | behöver till viss del fortsatt träning och  kunskap | behöver eventuellt klargöranden inom vissa  områden | behöver ingen ytterligare kunskap eller träning |

Vänligen ta ställning till följande påståenden:

Kunskap

|  | 1 | 2 | 3 | 4 | 5 |
| --- | --- | --- | --- | --- | --- |
| 1. En förståelse för möjligheter och begränsningar med digitala arbetssätt. |  |  |  |  |  |
| 2. En förståelse för mångfald och kulturella skillnaders roll i användningen av digitala  arbetssätt. |  |  |  |  |  |
| 3. En förståelse för hur det kliniska arbetet ska kvalitetssäkras*,* samt organisatoriska och  professionella riktlinjer, relaterade till digitala arbetssätt. |  |  |  |  |  |
| 4. En förståelse för informationshantering, samt juridiska aspekter vid insamling och  lagring av klientdata i användningen av digitala metoder. |  |  |  |  |  |
| 5. En förståelse av evidensbasen för digitala arbetssätt. |  |  |  |  |  |
| 6. En förståelse för frågor kring klinisk allians och engagemang vid digitalt klientarbete. |  |  |  |  |  |
| 7. En förståelse för de psykologiska verktyg som finns tillgängliga att administrera digitalt. |  |  |  |  |  |
| 8. En förståelse för digitala interventioner riktade till individer. |  |  |  |  |  |
| 9. En förståelse för digitala interventioner riktade till familjer, grupper eller organisationer. |  |  |  |  |  |
| 10. En förståelse för hur digitala arbetssätt påverkar kommunikationsprocesser  (exempelvis turtagning och användning av icke-verbal information). |  |  |  |  |  |
| 11. En förståelse av för- och nackdelar med onlinebaserade undervisningsmetoder och  digitala undervisningsprogram. |  |  |  |  |  |
| 12. En förståelse för hur handledning, ledarskap, och konsultationer behöver anpassas vid  digitala arbetssätt. |  |  |  |  |  |
| 13. Kunskap om digitala verktyg för att mäta kliniska utfall, monitorera, samt för utvärdering  av tjänster och forskning. |  |  |  |  |  |
| 14. En förståelse för klinisk säkerhet och riskhantering i samband med digitalt arbete på  distans. |  |  |  |  |  |
| 15. En förståelse för egna attityder, färdigheter, etiska principer och värderingar i  förhållande till digitala arbetssätt. |  |  |  |  |  |

Förmågor

|  | 1 | 2 | 3 | 4 | 5 |
| --- | --- | --- | --- | --- | --- |
| 1. Förmåga att respektera gränser och utveckla en positiv terapeutisk relation online. |  |  |  |  |  |
| 2. Förmåga att förmedla kulturellt anpassade psykologiska insatser i digitalt format,  inklusive genom tolkanvändning. |  |  |  |  |  |
| 3. Förmåga att följa organisatoriska och professionella riktlinjer avseende kvalitetssäkring  och informationshantering för digitala arbetssätt. |  |  |  |  |  |
| 4. Förmåga att förklara för- och nackdelar med digitala förmedlingssätt för klienter och få  deras informerade samtycke. |  |  |  |  |  |
| 5. Förmåga att genomföra korrekta kliniska risk- och säkerhetsbedömningar i  användningen av digitala arbetssätt. |  |  |  |  |  |
| 6. Förmåga att använda ett evidensbaserat förhållningssätt till att kritiskt utvärdera  digitala verktyg och interventioner för användning kliniskt och i forskning. |  |  |  |  |  |
| 7. Förmåga att introducera och engagera klienter till att använda digitala format. |  |  |  |  |  |
| 8. Förmåga att välja och administrera digitala psykologiska bedömningsverktyg utifrån  klientens behov. |  |  |  |  |  |
| 9. Förmåga att förmedla digitala psykologiska interventioner riktade till individer. |  |  |  |  |  |
| 10. Förmåga att förmedla digitala psykologiska interventioner riktade till familjer, grupper  eller organisationer. |  |  |  |  |  |
| 11. Förmåga att anpassa sin kommunikationsstil till klientens behov och det digitala format  som används. |  |  |  |  |  |
| 12. Förmåga att förmedla utbildande insatser i det digitala arbetet med klienter och  professionella (exempelvis psykoedukation och distansundervisning). |  |  |  |  |  |
| 13. Förmåga att genomföra handledning, arbeta i team och delta i digitala möten på  distans. |  |  |  |  |  |
| 14. Förmåga att digitalt samla in och hantera utfallsdata i kliniskt och forskningssyfte. |  |  |  |  |  |
| 15. Förmåga att reflektera över sina egna attityder, färdigheter, etiska överväganden och  värderingar i förhållande till digitala arbetssätt. |  |  |  |  |  |
| 16. Förmåga att upptäcka begränsningarna i sin egen kompetens inom digitala  arbetssätt. |  |  |  |  |  |
