# Supplementary material for: AI-Assisted Adaptation of the Swedish Version of the Digital Competencies for Applied Psychological Practitioners Scale: Preliminary Psychometric Evaluation
Source: JMIR Form Res. 2026 Sep 8;10:e92332. doi: 10.2196/92332 (PMC13552648; doi:10.2196/92332)
Supplement: Supplementary file 7 [file formative-v10-e92332-s007.docx]

Enkätstudie om kunskaper och färdigheter i digital klinisk psykologi

**Om forskningsprojektet och denna enkät**

Du inbjuds att delta i en enkätundersökning om kunskaper och färdigheter i digital klinisk psykologi. Studien syftar till att utvärdera en anpassad och översatt version av det engelska instrumentet *Digital Competencies for Applied Psychological Practitioners Scale* (DCAPP) som mäter psykologers förståelse och förmågor inom digital klinisk psykologi.

Insamlad data kommer att användas för att utveckla den översatta och anpassade versionen av DCAPP, samt undersöka instrumentets förmåga att mäta förändringar i kunskap och förmågor inom digital klinisk psykologi över tid. Data som samlas in kommer att delas med forskare vid Royal Holloway, University of London.

**Hur går studien till?**

Om du väljer att delta i studien innebär det att du fyller i en enkät med ett antal frågor digitalt vid två tillfällen med åtta veckors mellanrum. Enkäten beräknas ta cirka 15 minuter att fylla i vid varje enskilt tillfälle.

**Vad händer med mina data?**

Information som besvaras i enkäten kommer att förvaras i en krypterad databas och lagras i minst 10 år efter avslutad studie. Dina svar kommer att behandlas konfidentiellt och i avidentifierad form.

» Klicka här om du vill du veta mer om vad som händer med dina data.

Resultaten av studien kan komma att presenteras i vetenskapliga tidskrifter och konferenser. Om så är fallet kommer resultaten att publiceras på gruppnivå och ingen enskild deltagare kommer att identifieras.

Ansvarig för behandlingen av dina personuppgifter för studien är Linköpings universitet. Dina svar och dina resultat kommer att behandlas så att obehöriga inte kan ta del av dem. Enligt EU:s dataskyddsförordning (GDPR) har du rätt att kostnadsfritt få tillgång till de uppgifter om dig som hanteras i studien, och vid behov få eventuella fel rättade.

Du kan också begära att uppgifter om dig raderas och att behandlingen av dina personuppgifter begränsas.

Om du vill komma åt eller radera uppgifterna, vänligen kontakta dataskyddsombud kan nås via e-post dataskyddsombud@liu.se. Om du är missnöjd med hur dina personuppgifter behandlas har du rätt att lämna in ett klagomål till huvudansvarig forskare (se kontaktuppgifter nedan)

Om du är missnöjd med hur dina personuppgifter behandlas har du rätt att lämna in ett klagomål till Integritetsskyddsmyndigheten, som är tillsynsmyndighet.

Etikprövningsmyndigheten har bedömt att studien inte behöver tas upp till prövning eftersom ingen behandling av känsliga personuppgifter sker som kräver etikgodkännande enligt etikprövningslagen (Dnr 2025-00064-01).

**Hur får jag information om resultaten av studien?**

Kontakta nedanstående forskare:

Kristofer Vernmark

Leg psykolog och fil. dr vid Linköpings Universitet

E-post: [Kristofer.vernmark@liu.se](mailto:Kristofer.vernmark@liu.se)

**Försäkring och ersättning**

Ingen ersättning betalas ut. Inget ytterligare försäkringsskydd behövs för denna studie.

**Deltagandet är frivilligt**

Ditt deltagande är frivilligt och du kan när som helst välja att avbryta deltagandet. Redan insamlade data kommer dock att användas. Om du väljer att inte delta eller vill ställa in ditt deltagande behöver du inte ange varför, och det kommer inte heller att påverka din situation som anhörigvårdare eller dig som professionell i ditt arbete. Om du vill avbryta ditt deltagande, vänligen kontakta huvudansvarig forskare för studien (se nedan).

**Ansvarig för studien**

Kristofer Vernmark

Leg psykolog och fil. dr vid Linköpings Universitet

E-post: [Kristofer.vernmark@liu.se](mailto:Kristofer.vernmark@liu.se)

*Informerat samtycke via länk här*
