# Supplementary material for: AI-Assisted Adaptation of the Swedish Version of the Digital Competencies for Applied Psychological Practitioners Scale: Preliminary Psychometric Evaluation
Source: JMIR Form Res. 2026 Sep 8;10:e92332. doi: 10.2196/92332 (PMC13552648; doi:10.2196/92332)
Supplement: Supplementary file 8 [file formative-v10-e92332-s008.docx]

# Checklist 1 - Checklist for Reporting Results of Internet E-Surveys (CHERRIES)

| ***Checklist Item*** | ***Explanation*** |
| --- | --- |
| Describe survey design | Data were collected in an online survey between April and May 2025 using a convenience sampling method. Participants were fourth-year students in the five-year professional psychology master’s program at Linköping University |
| IRB approval | The Swedish Ethical Review Authority determined that the project did not involve processing of personal data as defined in Section 3 of the Swedish Ethical Review Act and the study did not require formal ethical review (Dnr 2025-00064-01). |
| Informed consent | Participation was voluntary and no incentives were provided for participation in the survey. All analyses were conducted on anonymized data.  Emails described the study and included a link to the online survey. Respondents were informed that the questionnaire was estimated to take about 15 minutes and that participation was anonymous. Invitations included a brief description of the study and a link to the survey. Participation was voluntary and participants were provided informed consent through the online survey platform and had to consent digitally before answering the questionnaire. Respondents could not access the survey if declining informed consent.  Respondents were informed that information provided in the survey will be kept in an encrypted database and stored for at least 10 years after completion of the study and that answers will be treated confidentially and in anonymized form. Information was provided that the survey was part of a research collaboration between Linköping University and Royal Holloway, University of London. |

| Data protection | The Iterapi platform used for this survey is a secure and widely used solution for providing online surveys, questionnaires, and internet-based interventions. The servers are physically located at Linköping University, Sweden, in a locked computer room accessible only to authorized personnel. A responsive website compatible with web browsers on mobile phones, tablets, and computers was set up to provide information about the study, including the possibility to register and provide informed consent. All communication between servers and users was encrypted, and the survey utilized its own dedicated database, configuration files, database passwords, and encryption keys. Survey data was stored in an encrypted format within the database, using advanced algorithms and secret keys to prevent unauthorized linkage between stored data and individual users. The servers, where the data was stored, were continuously monitored and updated following well-defined routines by the local security team at Linköping University. Access to user data was restricted to authorized members of the research team. The website and survey were open-access, meaning they were not password-protected and could be accessed by anyone with the survey link. To maintain anonymity, no client IP addresses were collected or stored. A cookie was used to prevent duplicate responses from the same browser and to recall partially saved answers if respondents completed the survey in multiple sessions. Respondents were able to revise their answers while completing the survey by using the Back and Forward buttons. Once the survey was submitted, answers could no longer be modified. There was no function for the participants to download their survey answers during or after the completion of the survey. |
| --- | --- |
| Development and testing | The survey was developed by researchers at Linköping University and included an adapted version of the DCAPP developed by researchers at Royal Holloway, University of London. The AI-assisted adaptation procedure is described in the manuscript and in appendices. The survey was administered in Swedish via the Iterapi platform, a secure and extensively used platform for collecting research data online, including questionnaires and surveys. The user experience of the online survey was tested by the webmaster.   The baseline questionnaire included the Swedish DCAPP 31-item scale and 11 additional questions on age, gender, previous digital training within the psychology master’s program or elsewhere, previous experience with delivering digital formats, and the perceived necessity of digital competencies as a clinical psychologist. The follow-up questionnaire included the DCAPP scale, as well as a question on whether the participants had received any additional training since commencing the digital psychology course. |

| Open survey versus closed survey | The website and survey were open-access, meaning they were not password-protected and could be accessed by anyone with the survey link. To maintain anonymity, no client IP addresses were collected or stored. A cookie was used to prevent duplicate responses from the same browser and to recall partially saved answers if respondents completed the survey in multiple sessions. |
| --- | --- |
| Contact mode | The study information and invitation to participate in the survey was distributed through email and the digital psychology course website. Emails with study information were sent to psychology master students in semester eight of their five-year program. Two reminders were sent at each measurement point. |
| Advertising the survey | No advertisement or paid online marketing channels were used. |
| Web/E-mail | The survey was web-based using a responsive website (with a dedicated URL) compatible with web browsers on mobile phones, tablets, and computers was set up to provide information about the study, including the possibility to register and provide informed consent. |

| Context | The website was created in Iterapi for the online survey with a dedicated URL not used for anything else than the survey. The link to the survey was provided in the information material. |
| --- | --- |
| Mandatory/voluntary | Participation in the online survey was voluntary. Respondents had to provide informed consent before starting the survey. |
| Incentives | No monetary incentives were offered, and respondents consented to provide their responses voluntarily. |

| Time/Date | Data collection took place between April and May 2025. |
| --- | --- |
| Randomization of items or questionnaires | Items were not randomized. |
| Adaptive questioning | The online questionnaire included free-text options for “yes” answers in the respondent characteristics section. E.g. Have you participated in any educational acitivity on digital psychology during the psychology masters program until now? If yes, describe the educational activity. |
| Number of Items | The baseline survey included the 31-item DCAPP scale and 11 additional items on age, gender, previous digital training within the psychology master’s program or elsewhere, previous experience with delivering digital formats, and the perceived necessity of digital competencies as a clinical psychologist.   The follow-up questionnaire included the DCAPP scale and a question on whether the participants had received any additional training since commencing the digital psychology course. Participants were also invited to provide feedback on each of the 31 DCAPP items, the comprehensibility of the Likert scale, and whether the knowledge and ability items were perceived as distinct constructs. |
| Number of screens (pages) | The baseline survey consisted of four webpages, including informed consent and a final page with repeated key information about the study.  The follow-up survey consisted of five webpages, including feedback on DCAPP items and the Likert-scale. |

| Completeness check | All items, except feedback on DCAPP items in the follow-up survey, were mandatory and selection of at least one response option was always enforced. At the end of the survey, respondents submitted the survey by clicking on the “submit” button. A submit date with a time stamp was saved in the platform. |
| --- | --- |
| Review step | Respondents were able to revise their answers while completing the survey by using the Back and Forward buttons. Once the survey was submitted, answers could no longer be modified. |
| Unique site visitor | To maintain anonymity, no client IP addresses were collected or stored. A cookie was used to prevent duplicate responses from the same browser and to recall partially saved answers if respondents completed the survey in multiple sessions. |
| View rate (Ratio of unique survey | Baseline: Information of the study was distributed to 55 students. No information was collected on number of visitors of the study website.  Follow-up: Information was distributed the 24 respondents who completed the baseline survey. No information was collected on number of visitors of the study website. |

| visitors/unique site visitors) |  |
| --- | --- |
| Participation rate (Ratio of unique visitors who agreed to participate/unique first survey page visitors) | Baseline: 24 provided informed consent, all of which started the survey. The participation rate was 100% (24/24).  Follow-up: 15 provided informed consent and 15 started the survey. The participation rate was 100% (15/15). |
| Completion rate (Ratio of users who finished the survey/users who agreed to participate) | Baseline: 24 respondents started the survey, and 24 completed it. The completion rate was 100% (24/24).  Follow-up: 15 respondents started the survey and 12 completed it. Non-completers only answered the first question in the survey. The completion rate was 80% (12/15). |
| Cookies used | A cookie was used to prevent duplicate responses from the same browser and to recall partially saved answers if respondents completed the survey in multiple sessions. |

| IP check | To maintain anonymity, no client IP addresses were collected or stored. |
| --- | --- |
| Log file analysis | No other techniques were used for identification of multiple entries. |
| Registration | The survey was open-access and did not require log in or registration. |
| Handling of incomplete  questionnaires | Only complete survey responses were analyzed. |

| Questionnaires submitted with an atypical timestamp | Timestamps were not used as a criterion. |
| --- | --- |
| Statistical correction | No such methods were used. |

This checklist has been modified from Eysenbach G. Improving the quality of Web surveys: the Checklist for Reporting Results of Internet E-Surveys (CHERRIES). J Med Internet Res. 2004 Sep 29;6(3):e34 [erratum in J Med Internet Res. 2012; 14(1): e8.]. Article available at [https://www.jmir.org/2004/3/e34/;](https://www.jmir.org/2004/3/e34/%3B) erratum available [https://www.jmir.org/2012/1/e8/.](https://www.jmir.org/2012/1/e8/) Copyright ©Gunther Eysenbach. Originally published in the Journal of Medical Internet Research, 29.9.2004 and 04.01.2012.

This is an open-access article distributed under the terms of the Creative Commons Attribution License ([https://creativecommons.org/licenses/by/2.0/),](https://creativecommons.org/licenses/by/2.0/)) which permits unrestricted use, distribution, and reproduction in any medium, provided the original work, first published in the Journal of Medical Internet Research, is properly cited.
